# Efficacy and Mechanism of Quercetin in the Treatment of Experimental Colitis Using Network Pharmacology Analysis

**DOI:** 10.3390/molecules28010146

**Published:** 2022-12-24

**Authors:** Qilian Zhang, Feifei Wen, Fang Sun, Zhengguang Xu, Yanzhan Liu, Chunxue Tao, Fei Sun, Mingchao Jiang, Mingtao Yang, Jing Yao

**Affiliations:** 1 School of Basic Medicine, Weifang Medical University, Weifang 261000, China; 2 School of Basic Medicine, Jining Medical University, Jining 272000, China; 3 School of Clinical Medicine, Qilu Medical University, Zibo 255000, China

**Keywords:** quercetin, ulcerative colitis, PI3K/AKT signaling, network pharmacology, molecular docking analysis

## Abstract

Quercetin, a flavonoid that is present in vegetables and fruits, has been found to have anti-inflammatory effects. However, the mechanism by which it inhibits colitis is uncertain. This study aimed to explore the effect and pharmacological mechanism of quercetin on dextran sodium sulfate (DSS)-induced ulcerative colitis (UC). Mice were given a 4% (*w/v*) DSS solution to drink for 7 days, followed by regular water for the following 5 days. Pharmacological mechanisms were predicted by network pharmacology. High-throughput 16S rDNA sequencing was performed to detect changes in the intestinal microbiota composition. Enzyme-linked immunosorbent assay and western blotting were performed to examine the anti-inflammatory role of quercetin in the colon. Quercetin attenuated DSS-induced body weight loss, colon length shortening, and pathological damage to the colon. Quercetin administration modulated the composition of the intestinal microbiota in DSS-induced mice and inhibited the growth of harmful bacteria. Network pharmacology revealed that quercetin target genes were enriched in inflammatory and neoplastic processes. Quercetin dramatically inhibited the expression of phosphorylated protein kinase B (AKT) and phosphatidylinositol 3-kinase (PI3K). Quercetin has a role in the treatment of UC, with pharmacological mechanisms that involve regulation of the intestinal microbiota, re-establishment of healthy microbiomes that favor mucosal healing, and the inhibition of PI3K/AKT signaling.

## 1. Introduction

Ulcerative colitis (UC) is an inflammatory bowel disease (IBD) with unclear pathogenesis. Its clinical symptoms include abdominal pain, diarrhea, and mucinous purulent bloody stool. Long disease duration and a high recurrence rate substantially impact the quality of life of patients [1]. Currently, salicylic acid and glucocorticoids are the most commonly used medications to treat UC, but they are associated with many adverse reactions, which restrict their long-term use [2]. Animal models of colitis frequently involve the administration of dextran sodium sulfate (DSS), which causes clinical and histological reactions resembling those seen in people with IBD [3,4,5,6].

Phosphatidylinositol 3-kinase (PI3K), a member of the intracellular lipid kinase family, can be divided into type I, II and III isoforms, of which type I plays a very important role in tumors [7]. Protein kinase B (PKB, as known as Akt), a serine/threonine kinase associated with protein kinase C, is a direct downstream target of PI3K [8,9]. The PI3K/AKT signaling pathway is critical for controlling the development and progression of inflammation [10], and it participates in the regulation and release of pro-inflammatory cytokines in the intestinal mucosa of UC patients [11]. Blocking the PI3K/Akt signaling pathway can reduce the release of cytokines, attenuate the inflammatory response, and achieve therapeutic outcomes in UC patients [10,12,13,14]. Further targeting of this pathway could lead to the creation of new UC medications.

Network pharmacology is a research method that combines pharmacology with information technology based on system biology, bioinformatics and high-throughput histology [15]. Because it utilizes a large amount of data to mine the targets of drugs and explore the interaction mechanism between the drugs and diseases, it is often used to explore the basic pharmacological effects of drugs on diseases and their mechanisms [16,17]. On the one hand, the multi-component characteristics of traditional Chinese medicine prescription have the advantages of multi-links and multi-targets; on the other hand, its material basis and mechanism of action are difficult to determine. Combining network pharmacology with traditional Chinese medicine prescription can give full play to the advantages of traditional Chinese medicine more effectively [18,19].

The pathogenesis of inflammatory bowel disease (IBD) is still unclear, but it is generally believed that the occurrence of IBD may be related to the imbalance of intestinal flora in individuals [20,21]. Short-chain fatty acids (SCFAs), such as acetate, propionate and butyrate, are important metabolites of the gut microbiome. SCFAs producing bacteria or SCFAs itself act on host cells by affecting intestinal immune response, gene expression, cell proliferation and host metabolism, thus maintaining intestinal homeostasis and inhibiting intestinal inflammation. Gut dysbiosis often decreases SCFA levels and may lead to inflammatory bowel diseases. The imbalance of intestinal flora, especially the decrease of butyric acid production bacteria caused the decrease of butyric acid concentration, leading to abnormal immune response, resulting in mucosal damage, and thus the submucosal non-specific inflammatory response [22,23,24,25,26,27,28]. Therefore, reconstructing intestinal microflora homeostasis and increasing SCFA levels are promising therapeutic approaches.

Quercetin (3,5,7,3′,4′-pentahydroxyflavone) is a flavonoid found in vegetables and fruits such as apples, onions, berries, green tea and black tea. It is reported that quercetin has antiulcer, antitumor, antioxidant and antihypertensive properties [29]. Indeed, quercetin alleviated the decreased body weight and histological destruction of colon tissue in a colitis model induced by DSS [30]. Quercetin increases the number of Treg cells while decreasing the activity of macrophages, neutrophils, and Th17 cells [31]. However, the mechanism of action of quercetin is complex, and many aspects of its efficacy to treat colitis are still unknown. In the present study, we explored the potential therapeutic effect of quercetin on DSS-induced UC using network pharmacology combined with 16S rDNA sequencing and examined the influence and pharmacological mechanism of quercetin on mice in vivo and in vitro.

## 2. Materials and Methods

### 2.1. Ethics Statement

All procedures and assays were approved by the Institutional Animal Care and Use Committee of Jining Medical University (2021-DW-ZR-019).

### 2.2. Reagents

DSS (molecular weight 36–50 kDa) was purchased from MP Biomedicals Inc. (Irvine, CA, USA). The 5-aminosalicylic acid (5-ASA) was purchased from Sigma-Aldrich (St. Louis, MO, USA). Quercetin was purchased from Shanghai Yuanye Bio-Technology Co., Ltd. (Shanghai, China). Enzyme-linked immunosorbent assay (ELISA) kits for mouse interleukin (IL)-6, IL-1β, and tumor necrosis factor (TNF)-α were purchased from BioLegend (San Diego, CA, USA). Hematoxylin and eosin (H&E) were purchased from Solarbio Science & Technology Co., Ltd. (Beijing, China). The supplier of diaminobenzidine was Solarbio Science & Technology Co., Ltd. (Beijing, China). The bicinchoninic acid protein assay kit was purchased from Thermo Fisher Scientific (Waltham, MA, USA). Antibodies against PI3K p85, IL-6 and β-actin were obtained from Affinity Biosciences (Cincinnati, OH, USA). Antibodies against IL-1β and TNF-α were purchased from Bioworld Technology (St. Louis Park, MN, USA). Antibodies against phospho-Akt (Ser473) and Akt (C67E7) were obtained from Cell Signaling Technology (Danvers, MA, USA). The anti-occludin antibody was obtained from Proteintech Group (Wuhan, China). Transwell inserts (pore size of 0.4 μm) were purchased from Corning Inc. (Kennebunk, ME, USA).

### 2.3. Cell Culture

Mouse colon epithelial cells (MCECs) were cultured in high-glucose Dulbecco’s modified Eagle’s medium (DMEM), supplemented with 10% fetal bovine serum (FBS), and 1% penicillin and streptomycin (P/S) in a humidified incubator of 5% CO_2_ at 37 °C.

### 2.4. Screening of Cellular Drug Delivery Concentrations

At the logarithmic growth stage, mouse colon epithelial cells (MCECs) were uniformly spread in 96-well plates at a growth density of 30%, and after 24 h of incubation, a blank group (no cells were inoculated), a control group, and quercetin administration groups with different concentrations (500, 250, 125, 62.5, 31.25, and 15.625 μM) were set up, with 6 replicate wells in each group. After 24 h of drug administration, each well was continued to incubate for 1 h after adding 10 μL of CCK-8 reagent. The absorbance (A) values of each group were measured at 450 nm by an enzyme marker, and the cell survival rate was calculated. Cell survival rate (%) = (A spiked − A blank)/(A control − A blank) * 100%, and the experiments described above were repeated three times. The effect of different concentrations of quercetin on the survival rate of MCEC cells varied greatly. It was found that 62.5, 31.25, and 15.625 μM of quercetin had no significant effect on the survival rate of MCEC cells for 24 h. Therefore, 62.5 μM was chosen as the quercetin administration condition.

### 2.5. Animals and Experimental Protocols

Jinan Pengyue Experimental Animal Breeding Co. Ltd. (Jinan, China) provided female BALB/c mice, which were 35–40 days old and 18–22 g in weight. An appropriate temperature and humidity were maintained in the rearing room, and a normal circadian rhythm was established to maintain the normal physiological activities of the mice. Through the Jining Medical University’s Animal Care Committees, the animal care and protocols were authorized. A total of 40 BALB/c mice were randomly allocated into four groups (*n* = 10/group): untreated control, DSS model, DSS + 5-ASA, and DSS + quercetin. Except for the control group, mice were given a 4% (*w/v*) DSS solution to drink for 7 days before being given regular water for the next 5 days [3]. From day 1 to day 12, mice in the two treatment groups were administered 5-ASA (40 mg/kg) or quercetin (100 mg/kg) daily by gavage, while mice in the blank control and DSS model groups were administered normal saline. All mice were sacrificed on day 13, and their organs and feces were collected. Colon tissues from mice were fixed in 4% paraformaldehyde for H&E staining. The remaining colon tissues were stored in liquid nitrogen for western blot analysis. Feces samples obtained from the intestinal sections were transferred to a sterile tube using sterile forceps, then quickly placed into liquid nitrogen and stored at −80 °C immediately for microbiota analysis.

### 2.6. Evaluation of Colitis

During the experiment, body weight changes, bloody stool, fecal character and mental status were observed daily [3]. The disease activity index (DAI) scoring criteria are shown in Table 1.

### 2.7. Macroscopic Assessment and Histological Analysis

Colons were removed, opened longitudinally, washed with phosphate-buffered saline, then fixed in 4% paraformaldehyde and embedded in paraffin. Embedded tissues were sliced into sections of 4 mm thickness using a microtome, and then stained with H&E using a conventional protocol [3,32]. The histological change scoring criteria are shown in Table 2.

### 2.8. ELISA

ELISA kits were used to assess the secretion of IL-1β, TNF-α and IL-6 from colon tissues and supernatants of mouse colon epithelial cell (MCEC) cultures following the manufacturer’s recommendations, as previously described [33]. Each experiment was performed three times. Cytokine levels are shown in pg·mL^−1^.

### 2.9. 16S rDNA Sequencing and Microbiota Analysis

Sequencing of 16S rDNA was performed using the following primer pair: forward (5′-AGRGTTTGATYNTGGCTCAG-3′) and reverse (5′-TASGGHTACCTTGTTAS GACTT-3′). Third-generation microbial diversity was based on the PacBio sequencing platform, and the marker gene was sequenced by single molecule real-time sequencing (SMRT Cell). The species composition of each sample was revealed by filtering, clustering or denoising the circular consensus sequence, and species annotation and abundance analysis as previously described [34]. The following analyses were carried out: annotation and taxonomy analysis of species, significant difference analysis, and diversity analysis (alpha and beta diversity). The names of the repository/repositories and accession number(s) can be found at: https://www.ncbi.nlm.nih.gov/ (accessed on 23 September 2022), PRJNA881733.

### 2.10. Network Pharmacology

Targets of quercetin were gathered in TCMSP [35] (https://old.tcmsp-e.com/tcmsp.php, accessed on 15 March 2022), PharmMapper [36] (http://www.lilab-ecust.cn/pharmmapper/, accessed on 15 March 2022), and Swiss Target Prediction System [37] (http://www.swisstargetprediction.ch/, accessed on 15 March 2022). Duplicates were removed, and the remainder were imported into the Universal Protein (Uniprot) database [38] to standardize the target names and ultimately obtain drug-related targets. Similarly, the targets of UC found by searching the Gene Cards database [39] (https://www.genecards.org/, accessed on 15 March 2022) and OMIM database [40] (http://www.omim.org, accessed on 15 March 2022) using the keyword “ulcerative colitis” were overlapped, de-duplicated, and imported into the Uniprot database to standardize the target names and obtain the final UC disease targets.

Quercetin-related and UC disease targets were imported into the Venny 2.1 online mapping tool platform (https://bioinfogp.cnb.csic.es/tools/venny/index.html, accessed on 15 March 2022) to obtain “drug-disease” common targets. Protein-protein interaction (PPI) data were obtained from the STRING v. 11.5 database [41] (http://cn.string-db.org, accessed on 15 March 2022), with the species limited to “Homo sapiens” and the cutoff confidence score set at >0.4. PPI networks were established and visualized using Cytoscape software [42] (http://cytoscape.org/ver.3.9.1, accessed on 16 March 2022). Following that, enrichment analyses were carried out using Metascape [43] (https//metascape.org/gp/index.html, accessed on 16 March 2022).

### 2.11. Molecular Docking

We downloaded the 3D structure of quercetin in structure data file format from the Pubchem database (https://pubchem.ncbi.nlm.nih.gov, accessed on 25 May 2022), converted it to “mol2” format by Open Babel 3.1.1 software, used AutoDockTools to add hydrogen, set as ligand, determine the torque center and select the torsion key, and exported to PDBQT format. The target protein name was then entered into the Protein Data Bank (PDB) database (https://www.rcsb.org/, accessed on 25 May 2022), from which a human protein with one or more co-crystalline ligands and a low “resolution” value crystal structure was selected, saved in PDB format, dehydrogenated using AutoDockTools, set as a receptor and exported to PDBQT format. We adjusted the GridBox parameters by AutoDock 4.2.6 software [44] until the box wrapped all the receptor molecules, used the blind docking method to find the active site, exported the grid point parameter file (GPF), ran Autogrid 4, set the docking parameters and algorithm for docking, ran Autodock4, and checked the results. The docking results were visualized using PyMOL 2.4.0 software. Finally, to obtain the docking scores, the proteins and compounds were uploaded to DockThor [45] (https://www.dockthor.lncc.br/v2/, accessed on 25 May 2022) for online molecular docking.

### 2.12. Co-Culture and Scratch Assay

Mice induced with 4% DSS solution for 5 days were sacrificed on day 6. Peritoneal macrophages (Mφs) were collected and cultured in Dulbecco’s modified Eagle’s medium. MCECs were plated in 6-well culture plates and incubated at 37 °C in a 5% CO_2_ incubator. Peritoneal macrophage cell suspensions were added to the upper chamber of a Transwell insert (pore size of 0.4 μm), transferred to the 6-well culture plates and co-cultured. The co-culture system was treated with quercetin (62.5 μM). Monolayers of the MCECs were scratched and observed at 0 and 24 h following treatment. The percentage of coverage was calculated.

### 2.13. Western Blotting

Protein expression of Akt, p-Akt, PI3K p85, IL-6, TNF-α, IL-1β, β-actin and occludin were examined in colon tissues and MCECs, using the previously described western blotting method [46].

### 2.14. Statistical Analysis

Using GraphPad Prism software (GraphPad Software Inc., Avenida, CA, USA). All results are presented as means ± standard deviation from triplicate experiments. Group means were compared using Student’s *t*-test (for normal distribution). The *p* values < 0.05 were recognized as statistically significant. Details of each type of statistical analysis are provided in the figure captions.

## 3. Results

### 3.1. Quercetin Attenuated DSS-Induced Colitis in Mice

**Figure 1:**
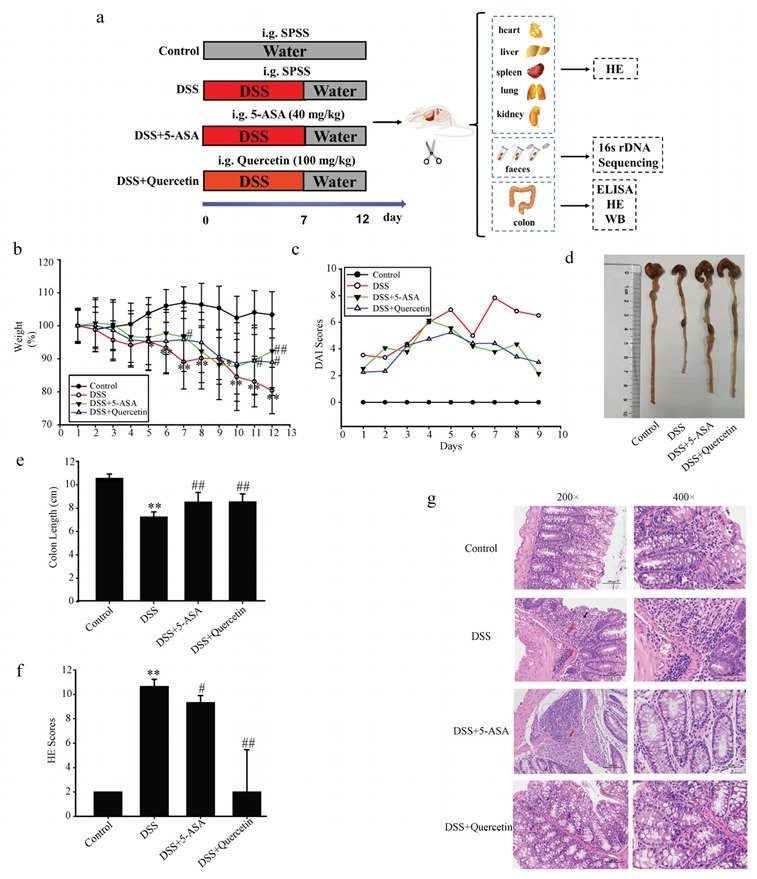
Quercetin reduces colon inflammation and damage induced by dextran sodium sulfate (DSS). (**a**) Flow chart of the experimental design. (**b**) Body weights of mice in the control, DSS, DSS + quercetin, and DSS + 5-aminosalicylic acid (5-ASA) groups. (**c**) Disease activity index. (**d**,**e**) Macroscopic appearance and the length of colons from each mouse group. (**f**) Histological changes. ** *p* < 0.01 compared with the control group; ^#^ *p* < 0.05, ^##^ *p* < 0.01 compared with the DSS group. (**g**) Hematoxylin and eosin staining of colonic sections. Infiltration of inflammatory cells in the mucosa or submucosa is indicated by black and red arrows, respectively.

To investigate the effects of quercetin on colitis, we added DSS to the drinking water of BALB/c mice for 7 days, followed by water treatment for 5 days. All animal procedures and assays are shown in Figure 1a. Mice in the DSS group showed substantial weight reduction compared with untreated control mice, which was improved after administration of quercetin (Figure 1b). The total DAI of DSS-induced mice was decreased by quercetin treatment, as evaluated by weight loss, and loose and bloody stools in the DSS + quercetin group (Figure 1c). In the process of modeling and administration, we observed the mental state of mice by naked eye, and found that the mental state of mice in the DSS group was poor and flagging, while the mental state of mice in the administration group was relatively good (data not shown). We also found that quercetin reversed the DSS-induced colon shortening (*p* < 0.01) (Figure 1d,e). Histopathological staining with H&E revealed that DSS treatment caused severe mucosal necrosis with submucosal congestion and edema, along with significant inflammatory cell infiltration. As compared with the 5-ASA treatment, this colonic damage and inflammatory cell infiltration were significantly attenuated by quercetin treatment (Figure 1f,g), which was consistent with the amelioration of colon edema and shortening.

### 3.2. Quercetin Inhibited the Secretion of Inflammatory Factors in Colonic Tissues of DSS-Induced UC Mice

**Figure 2:**
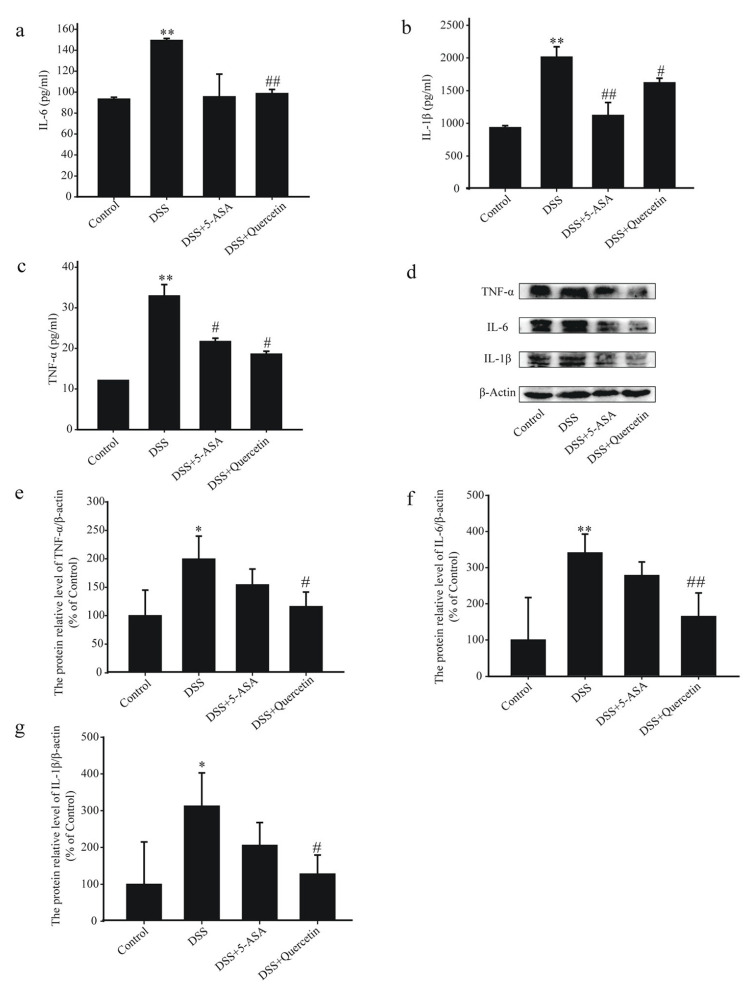
Quercetin reduces levels of inflammatory mediators in the colon of mice with dextran sodium sulfate (DSS)-induced colitis. Enzyme-linked immunosorbent assay analysis of interleukin (IL)-6 (**a**), IL-1β (**b**), and tumor necrosis factor (TNF)-α (**c**) concentrations in colonic tissue supernatants. (**d**–**g**) Western blotting analysis of TNF-α, IL-6 and IL-1β in colonic tissue extracts. * *p* < 0.05, ** *p* < 0.01 compared with the control group; ^#^ *p* < 0.05, ^##^ *p* < 0.01 compared with the DSS group.

DSS + quercetin-treated mice showed significantly reduced secretion of IL-6, IL-1β and TNF-α in colon tissues compared with DSS-treated mice (Figure 2a–c). Western blotting results in DSS + quercetin mice showed that quercetin inhibited the expression of TNF-α, IL-6 and IL-1β protein in colonic tissues compared with DSS-treated mice (Figure 2d–g).

### 3.3. The Herb-Ingredient-Target Network of Quercetin

**Figure 3:**
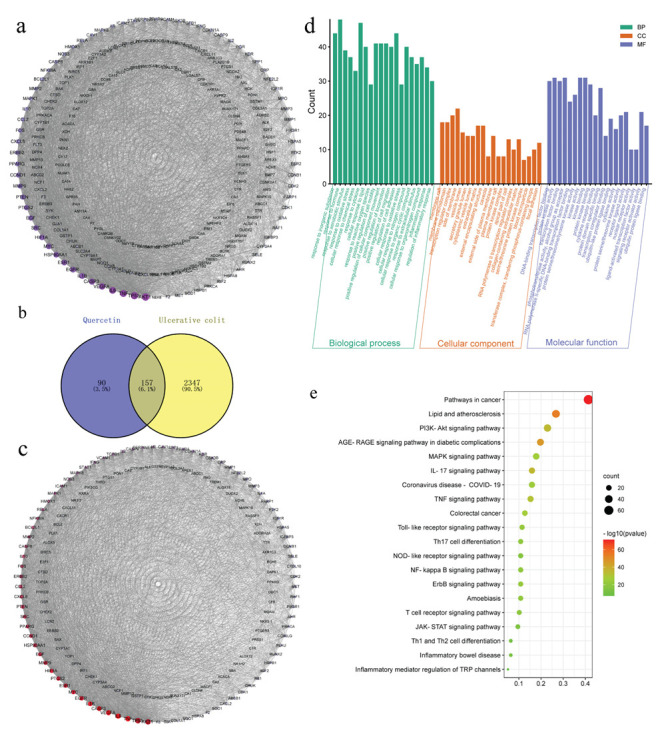
Network analysis of quercetin. (**a**) Protein-protein interaction (PPI) network diagram of quercetin-related proteins. (**b**) Venn diagram showing the intersection between ulcerative colitis (UC) and quercetin. (**c**) PPI interaction network of quercetin and UC intersection targets. Gene ontology (**d**) and Kyoto Encyclopedia of Genes and Genomes (KEGG) pathway enrichment (**e**) analysis.

Using the TCMSP, PharmMapper and Swiss Target Prediction databases, we identified 247 action targets of quercetin, including AKT, IL-6, TNF-α and IL-1β. Construction of a quercetin-related target interaction network with Cytoscape 3.9.1 software is shown in Figure 3a. The order is based on the degree value of importance of each action target. The degree value of the target increases with darker color and greater area.

Using “ulcerative colitis” as the keyword, searches of the GeneCards and OMIM databases yielded 4825 and seven potential targets of UC, respectively. After removing the duplicate targets, the remaining potential targets were standardized for gene names in UniProt, from which a total of 2504 potential UC targets were obtained. Using Venny 2.1, the 247 quercetin action targets were mapped with the 2504 UC disease targets on a Venn diagram, which revealed 157 common drug-disease targets (Figure 3b).

Next, the 157 common drug-disease targets were uploaded to the STRING database to build a PPI network, which included 157 nodes and 3157 edges. The topological properties of intersection target proteins were analyzed by Cytoscape software (Figure 3c), which found that the average degree of the network was about 40.2, the average betweenness was about 129.2, and the average closeness was about 0.00358. We found that there were 33 nodes, including betweenness and closeness, combined with the network diagram and topological attribute table, that were important targets of quercetin in UC (Table S1).

The 157 common drug-disease targets were also introduced into the Metascape platform for Gene Ontology (GO) biological function analysis and Kyoto Encyclopedia of Genes and Genomes (KEGG) pathway enrichment analysis. Taking *p* < 0.01 as the main screening standard, 2107 GO biological function entries were retrieved, including 1854 biological processes (BPs), 81 cellular components (CCs) and 172 molecular functions (MFs). A total of 202 signal pathways were obtained by KEGG pathway enrichment analysis (Figure 3d,e, and Table S2).

### 3.4. Quercetin Molecular Docking with the Top 10 Core Target Proteins in the PPI Network

**Figure 4:**
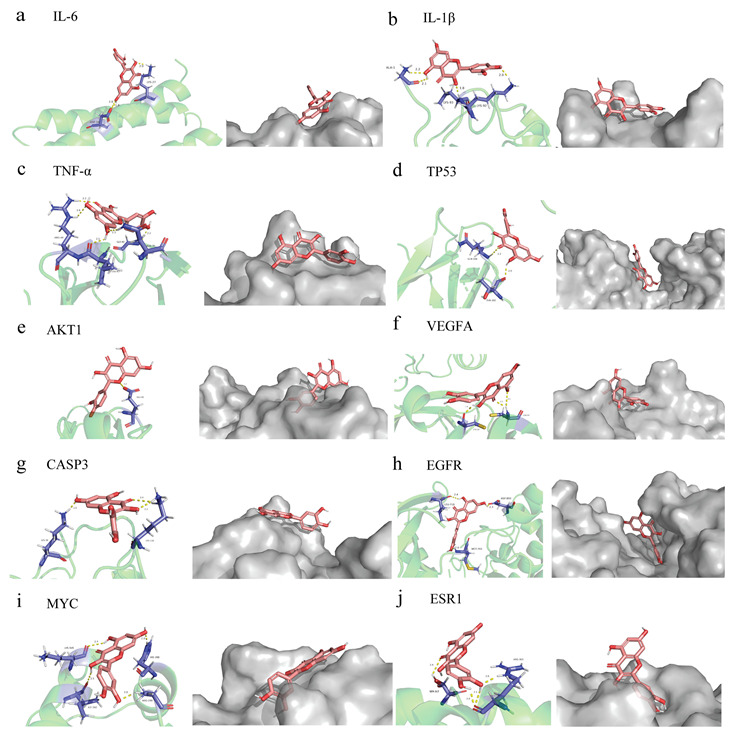
Partial diagram of molecular docking of quercetin with the following targets. (**a**) IL-6-quercetin; (**b**) IL-β-quercetin; (**c**) TNF-α-quercetin; (**d**) TP53-quercetin; (**e**) AKT1-quercetin; (**f**) VEGFA-quercetin; (**g**) CASP3-quercetin; (**h**) EGFR-quercetin; (**i**) MYC-quercetin; (**j**) ESR1-quercetin.

The affinity score in the molecular docking results reflects the level of binding between quercetin and the top ten core target proteins (Table S3). In general, the lower the affinity score, the more stable the binding conformation for ligand and receptor. Using AutoDock 4.2.6 software for molecular docking, we downloaded the results and related documents for quercetin and the following target proteins, taking the minimum binding energy as the reference index: AKT1 (PDB ID: 2uzs), TP53 (6ggb), TNF-α (2az5), IL-6 (1alu), VEGFA (5hhc), CASP3 (3deh), IL-1β (5r88), EGFR (2itv), MYC (6e16), and ESR1 (2qxs). The docking results indicated good binding ability between each of the ten target proteins and quercetin, with high potential biological activity (Figure 4a–j).

### 3.5. Fecal Microbiota Analysis

**Figure 5:**
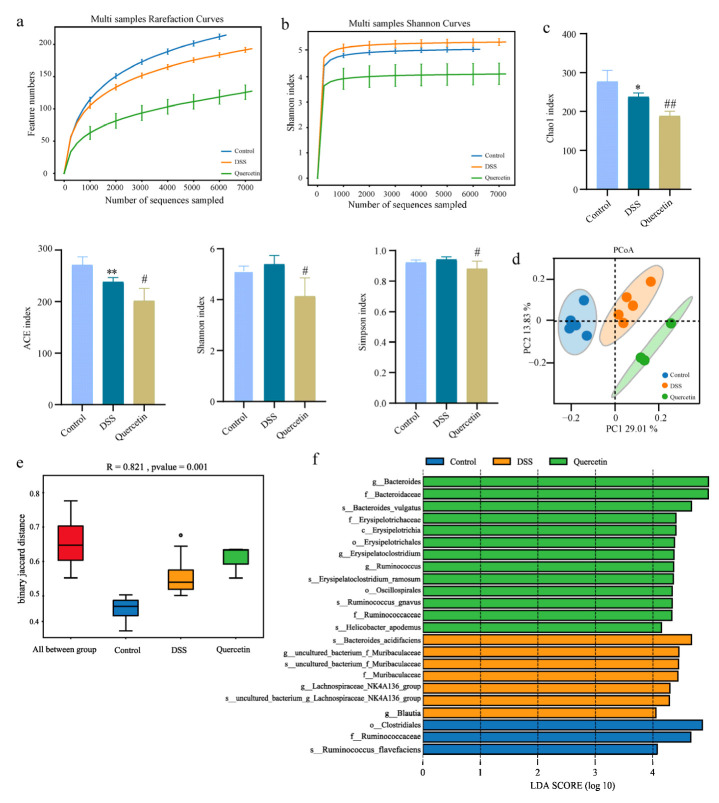
Alteration in microbiota profile in different groups. (**a**) Rarefaction analysis. (**b**) Shannon index curves. (**c**) Alpha-diversity index of microbial communities in mice treated with DSS or quercetin. * *p* < 0.05, ** *p* < 0.01 compared with the control group; ^#^ *p* < 0.05, ^##^ *p* < 0.01 compared with the DSS group. (**d**) PCoA plot of unweighted unifrac distance. (**e**) Anosim analysis of intestinal bacterial community in mice. (**f**) LEfSe analysis.

As the network pharmacological analysis revealed that quercetin had an antibacterial impact, we looked for changes in the microbiota composition. To determine the effect of quercetin on gut microbial composition, we performed 16S rDNA sequencing, which was evident from alpha and beta diversity estimation. Alpha diversity was evaluated using abundance indices (Chao1 and ACE) and diversity indices (Shannon and Simpson). The Chao1 and ACE estimates represent bacterial richness and species abundance, whereas Shannon and Simpson indices characterize the diversity of microorganisms. All sample libraries used in this study had coverage rates above 99%, indicating that the size of the library was adequate to include the vast majority of microorganisms. In all groups, the number of operational taxonomic units (OTU) reached saturation and appropriately represented the majority of species, and curve analysis including rarefaction curves and Shannon-Wiener curves was used to reflect the rationality of sample size (Figure 5a,b). The results showed that the Chao1 and ACE indexes in the quercetin group decreased compared with the model group, which indicated that the richness of species were decreased after drug administration. The Shannon and Simpson indexes were decreased by quercetin, indicating that the species diversity was decreased after drug administration (Figure 5c). Beta-diversity reflecting between-habitat diversity was calculated by unweighted unifrac. Principal Co-ordinates Analysis (PCoA) showed that the microflora of the groups were relatively in different areas, indicating that there were differences in the structure of intestinal microflora between the groups. The results suggested that the intestinal flora of mice was disturbed after modeling, and quercetin treatment could improve the intestinal flora disorder (Figure 5d). The non-parametric analysis of similarities (ANOSIM) analyses detected that the inter-group differences in community composition and abundance of the three groups were more pronounced than those within group (Figure 5e). In order to identify the bacterial groups with significant differences between the groups, linear discriminant analysis coupled with effect size measures (LEFSe) was performed. We found that compared with other groups, the abundance of bacteria including *Clostridiales*, *Ruminococcaceae* and *Ruminococcus flavefaciens* was the higher in control group (Figure 5f). The bacteria, including *Bacteroides acidifaciens*, *Muribaculaceae*, *Blautia* and the genus *Lachnospiraceae_NK4A136_group,* were markedly increased in DSS-treated group, which were *Bacteroidaceae*, *Erysipelotrichia*, *Oscillospirales*, and *Ruminococcaceae* in the quercetin-treated group (Figure 5f).

### 3.6. Quercetin Affected the PI3K-AKT Signaling Pathway in DSS-Induced Colitis

**Figure 6:**
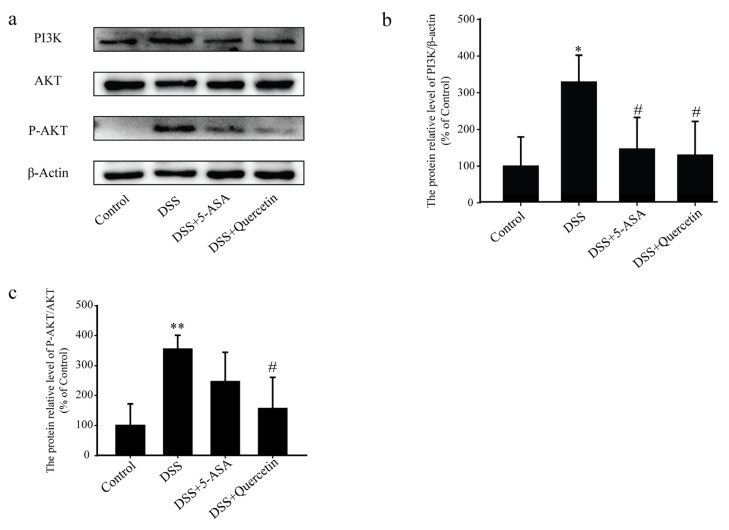
The anti-colitis effects of quercetin are mediated by inhibition of the dextran sodium sulfate (DSS)-induced phosphatidylinositol 3-kinase (PI3K)/protein kinase B (AKT) signaling pathway and the expression of inflammatory mediators. (**a**–**c**) Western blotting analysis of PI3K, AKT and phosphorylated (P)-AKT in colonic tissue extracts. * *p* < 0.05, ** *p* < 0.01 compared with the control group; ^#^ *p* < 0.05 compared with the DSS group.

Western blot analysis showed that treatment with quercetin halted the increased expression of PI3K and dramatically reduced the phosphorylation of AKT induced by DSS (Figure 6a–c). These results indicated that quercetin inhibited the activation of the PI3K-AKT signaling pathway to exert its anti-colitis effect.

### 3.7. Quercetin Suppressed Inflammation and Contributed to Mucosal Healing

**Figure 7:**
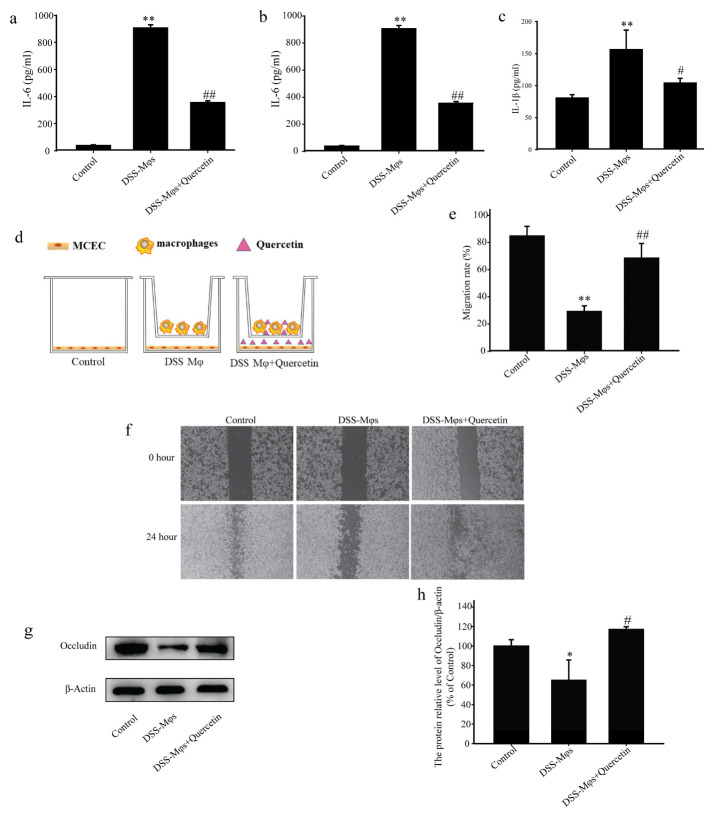
Quercetin suppressed inflammation and contributed to mucosal healing. Analysis of the concentrations of interleukin (IL)-6 (**a**), tumor necrosis factor (TNF)-α (**b**), and IL-1β (**c**) using an enzyme-linked immunosorbent assay. (**d**–**f**) Scratch assay showing the migration capacity of mouse colon epithelial cells (MCECs) co-cultured in vitro with macrophages under quercetin treatment. (**g**,**h**) Western blotting analysis of occludin expression in extracts of MCECs. * *p* < 0.05, ** *p* < 0.01 compared with the control group; ^#^ *p* < 0.05, ^##^ *p* < 0.01 compared with the DSS group.

To replicate the inflammatory microenvironment, we created a co-culture system using MCECs and Mφs. Peritoneal Mφs were extracted from DSS group mice and co-cultured with MCECs for 24 h. The concentrations of IL-6, TNF-α and IL-1β in the cell supernatants of the MCECs, as detected by ELISA assay, further suggested that quercetin significantly reduced the secretion of these inflammatory factors (Figure 7a–c).

In scratch experiments on the co-culture system, the capacity of MCECs to migrate was decreased in the presence of Mφs from DSS mice, in contrast to the promotion of MCEC migration by Mφs with quercetin-treated cells (Figure 7d–f). Western blot analysis showed that quercetin treatment significantly increased occludin expression, which was reduced in the DSS-Mφs group compared with that in the DSS-Mφs+Quercetin group (Figure 7g,h). These results indicated that quercetin attenuated DSS-induced downregulation of occludin to restore intestinal barrier function.

**Figure 8:**
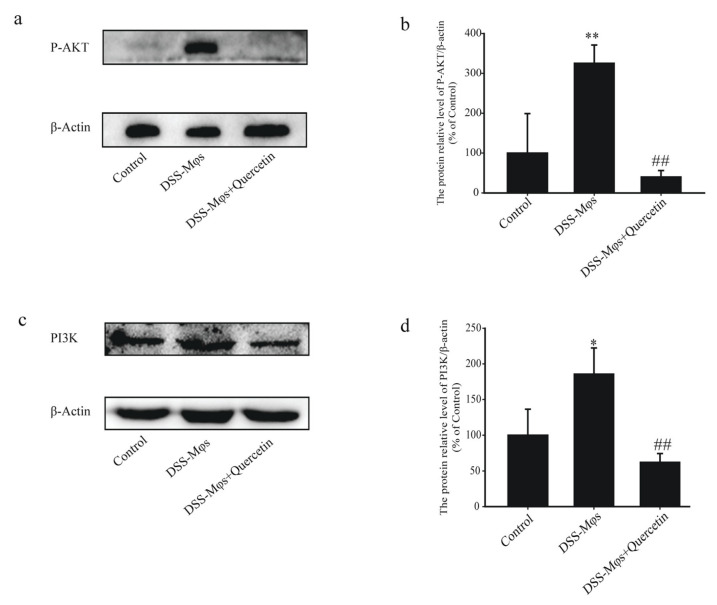
Quercetin inhibited the activation of the PI3K-AKT signaling pathway to exert an anti-colitis effect in vitro. (**a**–**d**) Western blotting analysis of phosphorylated (P)-AKT and PI3K expression in extracts of mouse colon epithelial cells. * *p* < 0.05, ** *p* < 0.01 compared with the control group; ^##^ *p* < 0.01 compared with the DSS group.

The western blot analysis of extracts of the MCECs also showed that, in the DSS-Mφs + Quercetin group, the overexpression of PI3K was halted and the phosphorylation of AKT induced by DSS was dramatically reduced (Figure 8a–d). These results further verified that quercetin inhibited the activation of the PI3K-AKT signaling pathway to exert an anti-colitis effect in vitro.

## 4. Discussion

In this study, we found that DSS-induced mice had serious inflammation and injury to colon tissues, with concomitant weight loss, bloody stools, loose stools and diarrhea, proving that the UC model was successful. All of these symptoms were improved by treatment with quercetin. Histopathological analysis indicated that DSS caused severe mucosal necrosis and submucosal edema, as well as significant inflammatory cell infiltration, all of which were significantly improved by quercetin, consistent with reduced inflammatory cell infiltration and secretion of inflammatory factors (IL-1β, TNF-α, IL-6).

Reportedly, the common flavonoid compound quercetin is the most effective scavenger of reactive oxygen species and prevents the synthesis of several pro-inflammatory substances, such as nitric oxide and TNF-α [47]. Prior to this study, the therapeutic effect of quercetin in UC had not yet been clarified, prompting us to perform a network pharmacological analysis of quercetin. A PPI topological analysis of 157 intersection genes revealed 33 strongly associated proteins. The results of molecular docking also verified that quercetin has superior affinities for the target genes *ESR1, IL-1β, TNF-α, IL-6, TP-53, VEGFA, CASP3, EGFR, MYC* and *AKT1*, and quercetin may exert powerful anticancer and anti-inflammatory effects via regulation of these targets.

**Figure 9:**
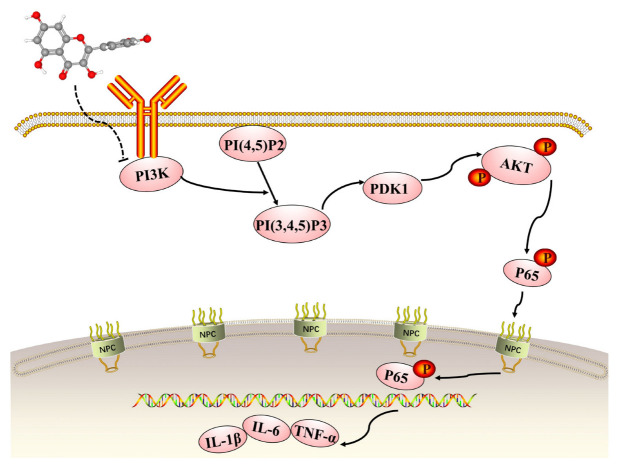
The role of the phosphatidylinositol 3-kinase (PI3K)/ protein kinase B (AKT) signaling pathway in quercetin’s inhibition of dextran sodium sulfate-induced ulcerative colitis.

The KEGG enrichment analysis of the quercetin-UC targets indicated several inflammation-related pathways: the IL-17, Toll-like receptor, PI3K/Akt, TNF, MAPK, NF-kappa B, NOD-like receptor, and JAK-STAT signaling pathways, T helper cell 17 differentiation, and inflammatory mediator regulation of transient receptor potential channels. The PI3K/AKT signaling pathway is recognized to be crucially important in inflammatory illnesses, especially IBD [10]. Quercetin has a role to play in the treatment of UC via inhibition of the PI3K/AKT signaling pathway, and its mechanism of action is shown in Figure 9. Upon activation of PI3K by multiple upstream cell surface receptors, type I PI3K catalyzes phosphatidylinositol 4,5-bisphosphate phosphorylation at the D3 position of the inositol ring to generate the second messenger phosphatidylinositol 3,4,5-trisphosphate (PIP3), which in turn activates PKB/AKT [7,48]. AKT and the upstream 3-phosphatidylinositol-dependent protein kinase-1 (PDK1) interacts with PIP3 through the pleckstrin-homology structural domain in PI3K and activates internal Thr308 site phosphorylation via PDK1 [49,50,51]. Upon activation of the PI3K/AKT pathway, IκBα is phosphorylated by IκB kinases (IKK) and then degraded by ubiquitin-mediated proteolysis, which promoted the phosphorylation and nuclear translocation of NF-κB p65 and further activated the expression of downstream inflammatory mediators [52,53,54,55]. In healthy colon tissues, IL-1β, TNF-α and IL-6 are expressed at low levels, but they are activated and upregulated during inflammation. Our western blotting results showed that quercetin inhibited the PI3K/AKT signaling pathway to exert anti-inflammatory effects, which validated the KEGG enrichment results. Meanwhile, it effectively enhanced the expression of occludin and lowered the expression of IL-1β, TNF-α and IL-6. Our in vitro experiments further demonstrated that quercetin could promote mucosal healing and inhibit the secretion of inflammatory factors as well as the PI3K/AKT signaling pathway.

The composition of the human gut microbiota is linked to health and disease. Dysbiosis reflects a change in the balance of the makeup of the gut microbiota, and increases the risk of developing IBDs including Crohn’s disease and UC [56]. *Bacteroidete*, the dominant flora in the colon, has attracted considerable attention [57]. It is reported that the relative abundance of *Bacteroides* in IBD patients is markedly lower than that in healthy participants [58,59]. A number of studies have shown that the abundant species of the common *Bacteroidetes*, including *Bacteroides vulgatus* and other key *bacteroidetes*, are beneficial to the recovery of intestinal health in patients with IBD, showing potential therapeutic potential [60,61]. In addition, *Erysipelotrichia*, *Erysipelotrichales*, *Erysipelotrichaceae*, *Oscillospirales* and *Ruminococcaceae* can produce SCFAs to protect the gut from damage and reduce the degree of colonic inflammatory injury, and the decrease in their relative abundance can lead to gastrointestinal disorders [62,63,64,65,66,67]. The results showed that the relative abundance of *Bacteroidaceae*, *Erysipelotrichia*, *Oscillospirales*, and *Ruminococcaceae* were significantly increased in the quercetin-treated group. Previous studies suggested that the relative abundance of *Lachnospiraceae* and *Lachnospiraceae_NK4A136_group* was significantly increased in colitis mice [68,69,70,71], which was consistent with our results. The results taken together indicated that quercetin effectively prevented the development and progression of experimental colitis by altering the composition of gut microbiota by increasing the abundance of beneficial bacteria and reducing the abundance of harmful bacteria.

The immune dysfunction of macrophage-driven intestinal microenvironment plays a crucial role in the pathological mechanism of UC; Mφs are highly plastic antigen-presenting cells that link the innate and adaptive immune systems, and macrophages can polarize into M1 type and M2 type with different functions in specific microenvironment. M1 type is an inflammatory type that releases ILs to stimulate inflammatory response, M2 type plays an anti-inflammatory role and can promote wound healing [72,73]. Animal lifeforms depend heavily on epithelial and/or endothelial barriers. An essential part of these barriers is the tight junction, of which occludin is a critical component [74]. To simulate the inflammatory environment surrounding epithelial cells, we established a co-culture system of MCECs and peritoneal Mφs extracted from DSS group mice for scratch assays. Peritoneal Mφs were extracted from DSS group mice and co-cultured with MCECs for 24 h. The DSS-Mφs + quercetin group was treated with quercetin (62.5 μM) on the basis of the DSS-Mφs group, and a blank control group of MCECs was not co-cultured with Mφs. While the DSS-Mφs group inhibited the migration of MCECs, no such effect was seen in the DSS-Mφs + quercetin group. It has been proved that quercetin can inhibit inflammatory reaction and promote wound healing by promoting the transformation of macrophages from M1 phenotype to M2 phenotype [75]. Therefore, we speculate that quercetin may promote M1 Mφs to M2 or impede the transition to M1 Mφs, thereby reducing the level of proinflammatory ILs in DSS induced colitis mice and promoting mucosal healing.

## 5. Conclusions

The unclear etiology and pathogenesis of UC have created urgency in the search for new and effective treatments. Our study substantiates a role for quercetin in the treatment of UC via inhibition of PI3K/AKT signaling, restoration of the intestinal barrier, and regulation of the gut microbiota, with no obvious tissue damage or side effects in mice. We propose that quercetin might be a feasible treatment option for UC and could be developed as a new therapeutic agent.

## Figures and Tables

**Table 1:** Scoring method of the disease activity index in the four study groups.

Score	Weight Loss (%)	Stool Consistency	Occult Blood
0	0	Normal	Normal
1	1–5	-	+
2	5–10	Loose Stool	++
3	10–15	Pasty stool	+++
4	>15	Diarrhea	+++

**Table 2:** Scoring criteria for histological changes.

Score	Number of Ulcers	Epithelial Cell Changes	Inflammatory Infiltration
0	0	Normal	Normal
1	1	Goblet cell deletion	Pericrypt infiltration
2	2	Goblet cell large area deletion	Mucosal muscularis infiltration
3	3	Crypt absence	Mucosal muscularis Large area infiltration
4	>3	Crypt Large area absence/polypoid regeneration	Submucosal infiltration

## Data Availability

The datasets presented in this study can be found in online repositories. The names of the repository/repositories and accession number(s) can be found at: https://www.ncbi.nlm.nih.gov/ (accessed on 23 September 2022), PRJNA881733.

## Supplementary Materials

The following supporting information can be downloaded at: https://www.mdpi.com/article/10.3390/molecules28010146/s1, Table S1: topological analysis results of main target network; Table S2: the enrichment pathways corresponding to intersection genes; Table S3: the affinity scores of quercetin with the top ten targets.

## Abbreviations

DSS: dextran sodium sulfate; UC, ulcerative colitis; 5-ASA, 5-aminosalicylicacid; HE, hematoxylin and eosin; TNF-α, tumor necrosis factor alpha; IL-1β, interleukin 1 beta; IL-6, interleukin 6; Mφs, macrophages; IBD, inflammatory bowel disease; MCEC, mouse colon epithelial cell; Uniprot, universal protein; PPI, protein-protein interaction.
