# Supplementary material for: Efficacy and Mechanism of Quercetin in the Treatment of Experimental Colitis Using Network Pharmacology Analysis
Source: Molecules. 2022 Dec 24;28(1):146. doi: 10.3390/molecules28010146 (PMC9822290; doi:10.3390/molecules28010146)
Supplement: Supplementary file 1 [file molecules-28-00146-s001.zip › molecules-2046578-supplementary.pdf]

Table S1. Topological analysis results of main target network.

Targets	Degree	Betweenness	Closeness
TP53	117	0.055373284	0.795918367
AKT1	117	0.04963017	0.795918367
TNF	112	0.046052317	0.776119403
IL6	110	0.041614556	0.768472906
VEGFA	109	0.03820015	0.76097561
CASP3	102	0.024361759	0.735849057
IL1B	99	0.02340656	0.728971963
EGFR	95	0.026241713	0.71559633
MYC	94	0.019600855	0.712328767
ESR1	93	0.025129339	0.705882353
PTGS2	92	0.026761069	0.705882353
HIF1A	92	0.013973436	0.702702703
MMP9	89	0.026755905	0.693333333
EGF	87	0.017712971	0.68722467
HSP90AA1	85	0.024082187	0.684210526
CCND1	84	0.010874663	0.67826087
PTEN	82	0.014592393	0.672413793
SRC	82	0.008720338	0.672413793
PPARG	82	0.026125668	0.672413793
CXCL8	79	0.008244547	0.663829787
ERBB2	77	0.019298793	0.658227848
CCL2	77	0.009824196	0.655462185
FOS	76	0.009270428	0.655462185
IL10	73	0.007278643	0.647302905
CASP8	71	0.008450925	0.639344262
BCL2L1	69	0.005797696	0.634146341
RELA	65	0.005690601	0.626506024
HMOX1	65	0.005732155	0.624
MAPK1	63	0.006975278	0.621513944
SERPINE1	57	0.00558362	0.604651163
AR	56	0.005933897	0.6
PPARA	54	0.012369688	0.6
CRP	50	0.010112801	0.590909091

Table S2. The enrichment pathways corresponding to intersection genes.

Description	Count	Pvalue
Pathways in cancer	65	$4.17\times10\text{-}^{72}$
Lipid and atherosclerosis	42	1.31×10^{-54}
AGE-RAGE signaling pathway in diabetic complications	31	3.27×10^{-47}
IL-17 signaling pathway	25	3.21×10^{-36}
PI3K-Akt signaling pathway	36	5.43×10^{-36}
TNF signaling pathway	24	2.81×10^{-32}
Colorectal cancer	20	8.06×10^{-28}
MAPK signaling pathway	28	3.21×10^{-27}
Coronavirus disease - COVID-19	25	1.01×10^{-25}
Toll-like receptor signaling pathway	18	1.27×10^{-22}
ErbB signaling pathway	17	1.42×10^{-22}
Amoebiasis	17	3.97×10^{-21}
NF-kappa B signaling pathway	17	5.64×10^{-21}
Th17 cell differentiation	17	1.11×10^{-20}
T cell receptor signaling pathway	16	2.31×10^{-19}
NOD-like receptor signaling pathway	17	1.20×10^{-16}
JAK-STAT signaling pathway	15	7.78×10^{-15}
Inflammatory bowel disease	10	1.50×10^{-12}
Th1 and Th2 cell differentiation	10	5.38×10^{-11}
Inflammatory mediator regulation of TRP channels	8	4.70×10^{-08}

Table S3. The affinity scores of quercetin with the top 10 targets.

Targets	Affinity
TP53	-7.438
AKT1	-6.724
TNF-α	-7.401
IL-6	-7.318
VEGFA	-7.42
CASP3	-7.535
IL-1β	-7.599
EGFR	-6.91
MYC	-6.752
ESR1	-9.298
